# AI-assisted versus manual sustainability assessment of a high-throughput LC–MS/MS method for psychotropic and OTC drugs of abuse in human plasma

**DOI:** 10.1038/s41598-026-59794-z

**Published:** 2026-07-06

**Authors:** Hend Z. Yamani, Khaled Hesham, Shereen M. Tawakkol, Lobna A. Hussein, Nesma M. Fahmy

**Affiliations:** 1 Pharmaceutical Analytical Chemistry Department, Faculty of Pharmacy, Ain Shams University, Cairo, 11566 Egypt; 2 Pharmaceutical Chemistry Department, Faculty of Pharmacy, Ahram Canadian University, Giza, Egypt

**Keywords:** Dextromethorphan, Pseudoephedrine, Fluoxetine, Olanzapine, LC–MS/MS, AI-assisted, Biological techniques, Chemistry, Drug discovery

## Abstract

A rapid, sensitive, and selective LC–MSMS method was developed and validated for the quantification of dextromethorphan (DXM), pseudoephedrine (PSE), olanzapine (OLA), and fluoxetine (FLU) in human plasma. Mixture 1 (DXM/PSE) and mixture 2 (OLA/FLU) are fixed-dose combinations commonly misused at high doses for their euphoric effects. The proposed method employed a simple protein precipitation technique for sample preparation, using a cost-effective cross-over internal standard strategy; OLA for mixture 1 and DXM for mixture 2. Chromatographic separation was achieved on a Hypersil GOLD column (100 × 3 mm, 1.9 µm) using an isocratic mobile phase consisting of acetonitrile and 0.1% formic acid (70:30, v/v) at a flow rate of 0.3 mL/min. The short runtime of 2.5 min enables high-throughput analysis. Detection was performed in positive ionization mode using multiple reaction monitoring (MRM). The method exhibited linearity over concentration ranges of 0.05–25.0 ng/mL for DXM, 2.0–1000.0 ng/mL for PSE, 0.2–20.0 ng/mL for OLA, and 0.5–50.0 ng/mL for FLU with lower limits of quantification (LLOQs) of 0.05, 2.0, 0.2, and 0.5 ng/mL, respectively. The method was successfully validated in accordance with FDA and ICH bioanalytical method validation guidelines, demonstrating satisfactory selectivity, accuracy, and precision. The validated method demonstrated high extraction recovery (> 90%), limited, reproducible matrix effects (IS-normalized matrix factor CV ≤ 15%). This study represents a novel application of artificial intelligence (AI)-assisted evaluation, utilizing a universally accessible model to assess the greenness and whiteness of the proposed LC–MS/MS method through the Auto-AGREE and Auto-RGB 12 frameworks. The AI-generated assessments demonstrated high agreement with traditional metrics, highlighting the potential of AI tools to provide rapid and objective holistic sustainability evaluations for the global analytical community.

**Supplementary Information:**

The online version contains supplementary material available at 10.1038/s41598-026-59794-z.

## Introduction

Dextromethorphan (DXM), a synthetic morphinan derivative, is a widely used cough suppressant^1^, possessing a molecular formula of C_18_H_25_NO, and a molecular weight of 271.40 g/mol^2^. Pseudoephedrine (PSE) is a sympathomimetic drug belonging to the class of substituted amphetamines. As a potent nasal decongestant, it functions by constricting blood vessels in the nasal passages^3^, with a molecular formula of C_10_H_15_NO, and a molecular weight of 165.23 g/mol^4^. Due to their complementary therapeutic effects, DXM and PSE are commonly co-formulated in over-the-counter and prescription medications to relieve symptoms of the common cold and cough^5^.

After oral administration, DXM is rapidly absorbed and undergoes extensive first-pass metabolism, predominantly via the cytochrome P450 2D6 (CYP2D6) pathway. Following a single 30 mg oral dose in extensive metabolizers, the reported maximum plasma concentration (C_max_) is approximately 2.9 ng/mL^6^. Similarly, PSE is rapidly and completely absorbed after oral dosing; A single 30 mg dose produced a geometric mean C_max_ of 98.5 ng/mL^7^.

While these drugs are widely used in clinical practice for the management of cough and cold symptoms, their misuse have been documented in the literature. At high doses, DXM can be misused for its psychoactive, dissociative effects^8^, while pseudoephedrine is a key precursor in the illicit synthesis of methamphetamine in addition to its abuse potential^9^. This highlights the need for analytical methods that may support clinical and forensic investigations. Although several methods have been reported for the analysis of each drug individually, a reliable, single-run method for the simultaneous quantification of both compounds is highly desirable. A review of the literature revealed that methods reported for the simultaneous determination of DXM and PSE are primarily limited to pharmaceutical preparations such as tablets and syrups, employing techniques like HPLC^10,11^ or spectrophotometry^12,13^. While effective for quality control in drug formulations, these methods often lack the sensitivity and selectivity required for the analysis of low drug concentrations in complex biological matrices like human plasma. Consequently, reports describing dedicated LC–MS/MS methods for the simultaneous determination of DXM and PSE in human plasma remain very limited despite their frequent co-administration and abuse potential. This represents a significant bioanalytical gap, particularly for clinical and forensic studies that require the simultaneous determination of both compounds.

The fixed-dose of olanzapine (OLA) and fluoxetine (FLU) combination is prescribed to treat depression associated with bipolar disorder, and treatment-resistant depression^14^. OLA, with a molecular formula of C_17_H_20_N_4_S, and a molecular weight of 312.4 g/mol^15^, typically reaches a maximum concentration (C_max_) of up to 12 ng/mL at steady state after 10 mg dose^16^. while fluoxetine which has a molecular formula of C_17_H_18_F_3_NO, and a molecular weight of 309.33 g/mol^17^, has a C_max_ of about 11.8 ng/mL following a single oral 20-mg dose^18^.

The substantial inter-individual variability in olanzapine plasma concentrations, together with the well-established concentration–response relationship, underscores the clinical importance of therapeutic drug monitoring (TDM). According to the Arbeitsgemeinschaft für Neuropsychopharmakologie und Pharmakopsychiatrie guidelines, TDM is strongly recommended for olanzapine^19^. In the case of fluoxetine, TDM is considered useful, particularly due to its long half-life prolongation. Furthermore, there is potential for their intentional abuse^20–22^. Therefore, the simultaneous determination of these compounds is not only relevant for TDM but also for toxicological and forensic applications, particularly in cases of intentional misuse or overdose.

Although simultaneous determination of OLA along with FLU has been reported using various analytical techniques including HPLC^23–25^ and spectrophotometry^26,27^, these methods were primarily developed for pharmaceutical dosage forms and have limited applicability to biological matrices. LC–MS/MS methods for the simultaneous quantification of OLA and FLU in human plasma have been described^28–32^.

The common thread linking two pharmacologically distinct binary mixtures is their documented potential for misuse, abuse, and presence in toxicological workflows. This selection was made to evaluate the performance of a unified LC–MS/MS method to accommodate analytes with different pharmacological classes, and physicochemical properties, but similar relevance in clinical and forensic contexts under a single set of conditions. This approach highlights the versatility of a single high-throughput LC–MS/MS platform for dual application in clinical therapeutic drug monitoring and forensic/toxicological analysis, enabling routine laboratories to efficiently handle compounds with varying physicochemical characteristics.

In this work, a novel unified LC–MS/MS platform is proposed for the determination of two binary mixtures (DXM/PSE and OLA/FLU) in human plasma under a single set of chromatographic conditions. From an operational perspective, this unified approach eliminates the need for method switching, thereby reducing instrument downtime, streamlining analytical workflows, and enhancing laboratory efficiency in both clinical and forensic settings. The method validation was performed in accordance to FDA and ICH bioanalytical method validation guidelines^33,34^.

**Fig. 1:**
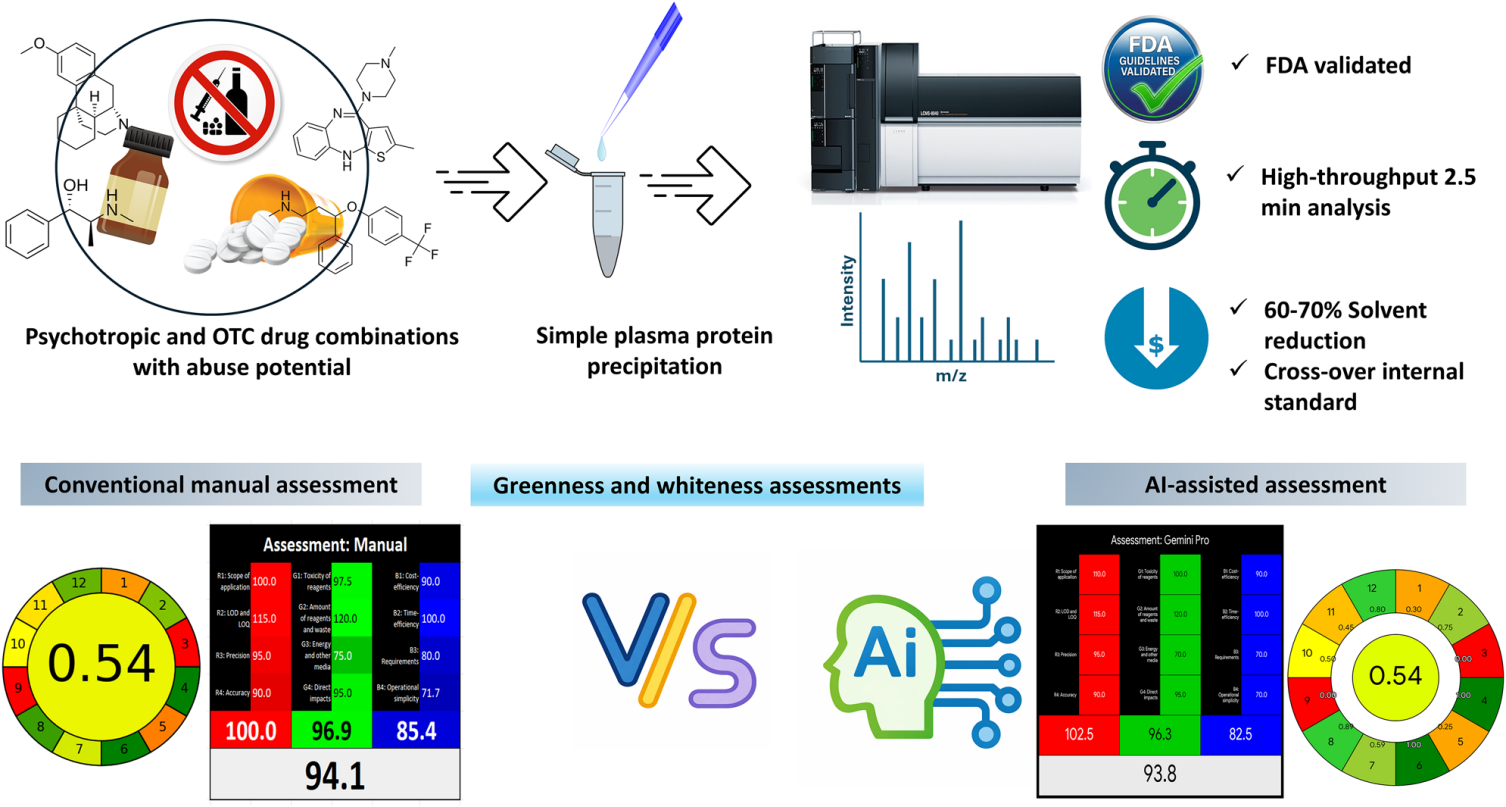
Integrated experimental and evaluative framework for high-throughput bioanalysis and AI-assisted sustainability assessment.

Artificial intelligence (AI) has increasingly been explored in analytical chemistry to enhance data processing and decision-support analysis. While AI is frequently cited for its potential in optimizing experimental conditions and enhancing peak detection^35,36^, t the present study introduces, for the first time, a comparative evaluation of AI-assisted (Auto-AGREE and Auto-RGB12) and conventional (AGREE and RGB12) metrics for assessing the greenness and whiteness of the newly developed LC–MS/MS method. This comparative study aims to evaluate the agreement between AI-assisted and conventional sustainability assessment approaches. By demonstrating the potential of AI-assisted assessment using models such as Gemini Pro to deliver objective, and rapid, this work highlights how structured AI-assisted evaluation can streamline sustainability assessment for the analytical community. The integrated experimental and evaluative framework proposed in this study is summarized in Fig. 1.

## Experimental

### Chemicals and reagents

DXM, PSE, FLU, and OLA were supplied by October Pharma Pharmaceutical Company (Egypt). According to the supplied certificates, the purities were 100.56 ± 0.85 for PSE, 100.22 ± 0.65 for DXM, 100.25 ± 0.55 for FLU and 100.72 ± 0.85 for OLA. LC–MS grade water, acetonitrile and formic acid were purchased from Sigma-Aldrich (Germany). Blank drug-free human plasma was purchased from VACSERA (Giza, Egypt) and was kept frozen at − 20 °C until used. The plasma was supplied as anonymized, de-identified samples. As no direct human participation was involved, ethical approval and informed consent from the authors were not required.

### Liquid chromatographic and mass spectrometric conditions

The method was applied to two distinct binary mixtures: (1) DXM and PSE with OLA as the internal standard (IS), and (2) OLA and FLU with DXM as the IS. Chromatographic separation was performed using a Shimadzu Nexera-i LC-2040C 3D Plus system (Kyoto, Japan). Separation of analytes was achieved on a Hypersil Gold column (100 mm × 3 mm, 1.9 μm). The mobile phase consisted of 0.1% aqueous formic acid and acetonitrile (30:70, v/v) delivered isocratically at a constant flow rate of 0.3 mL/min. The column temperature was set at 30°C. The complete runtime for a single injection was 2.5 min.

For detection, the liquid chromatography system was coupled to a Shimadzu LCMS-8040 triple quadrupole mass spectrometer (Kyoto, Japan). Mass spectrometric analysis was conducted using an electrospray ionization (ESI) interface operating in positive ion mode. Quantification was performed via multiple reaction monitoring (MRM), with the following precursor-to-product ion transitions: DXM (*m/z* 271.9 → 215.1), PSE (*m/z* 166.25 → 148.1), OLA (*m/z* 312.9 → 256.05), and FLU (*m/z* 309.85 → 148.1). The optimized mass spectrometric parameters for all analytes are summarized in Table S1. Instrument control and data acquisition were managed using LabSolutions LCMS software.

### Procedure

#### Preparation of stock and working solutions

##### Mixture 1 (DXM/PSE)

Separate stock solutions of DXM, PSE, and the IS (OLA) were prepared in acetonitrile at a concentration of 200 µg/mL. These solutions were stored at 4°C. Working solutions were subsequently prepared by serially diluting the stock solutions with acetonitrile. The final concentration range for the working solutions was 0.5–250.0 ng/mL for DXM and 20.0–10,000.0 ng/mL for PSE. A separate working solution of the IS was prepared at a concentration of 75 µg/mL.

##### Mixture 2 (OLA/FLU)

Stock solutions of OLA, FLU and DXM (200 µg/mL) in acetonitrile were separately prepared and kept at 4°C. Then working solutions were prepared by serially diluting the stock solutions with acetonitrile. The working solutions concentration ranges for OLA and FLU were 2.0–200 ng/mL and 5.0–500 ng/mL respectively. The IS working solution used was 5 µg/mL.

#### Preparation of calibration standards (CSs) and quality control (QC) samples

Prior to analysis, frozen plasma samples ( − 20 °C) were thawed at room temperature. Both CSs and QC samples were prepared by spiking 50 µL each of the analyte and the IS working solutions into 350 µL of blank human plasma at the required concentration levels, yielding final volume of 500 µL, followed by sample processing.

The analytical sample set included a blank matrix sample (processed without any analytes or IS), a zero sample (containing only the IS), and a series of non-zero calibration standards to establish the analytical range, including the LLOQ.

##### Mixture 1 (DXM/PSE)

The non-zero CSs were prepared by spiking drug-free human plasma with increasing concentrations of DXM and PSE to yield final concentrations of: 0.05, 0.25, 0.5, 2.5, 5.0, 10.0 and 25.0 ng/mL for DXM and 2.0, 10.0, 20.0, 100.0, 200.0, 400.0 and 1000.0 ng/mL for PSE.

Additionally, QC samples were prepared at four concentration levels (LLOQ, Low (LQC), Medium (MQC), and High (HQC)) for DXM and PSE as follows: LLOQ-QC (0.05 ng/mL for DXM; 2 ng/mL for PSE), LQC (0.15 ng/mL for DXM; 6 ng/mL for PSE), MQC (7.5 ng/mL for DXM; 300 ng/mL for PSE), and HQC (20 ng/mL for DXM; 800 ng/mL for PSE). The IS (OLA), was added to a final concentration of 7500 ng/mL. This concentration was selected to ensure stable and reproducible signal intensity across the calibration range and effective correction for matrix-related variability while avoiding detector saturation.

##### Mixture 2 (OLA/FLU)

The non-zero CSs were prepared by spiking blank human plasma with increasing concentrations of OLA and FLU to yield the following final concentrations: 0.2, 0.5, 1.0, 3.0, 8.0, 15.0, and 20.0 ng/mL for OLA, and 0.5, 1.0, 2.5, 7.0, 18.0, 30.0, and 50.0 ng/mL for FLU.

Additionally, QC samples were prepared at four concentration levels for OLA and FLU: LLOQ (0.2 ng/mL for OLA; 0.5 ng/mL for FLU), LQC (0.6 ng/mL for OLA; 1.5 ng/mL for FLU), MQC (5 ng/mL for OLA; 15 ng/mL for FLU), and HQC (16 ng/mL for OLA; 40 ng/mL for FLU). The IS (DXM) was added to a final concentration of 500 ng/mL.

#### Pre-treatment of plasma

Protein precipitation method was employed to prepare plasma samples for analysis. A 0.5 mL aliquot of the plasma sample was transferred to a centrifuge tube, and protein precipitation was induced by the addition of 1.0 mL of acetonitrile, followed by two minutes of vortexing. The samples were then centrifuged for ten minutes at 5000 rpm. The resulting clear supernatant was collected, and a 10 µL aliquot was directly injected into the LC–MS/MS system without further cleanup or dilution.

### Method validation

Bioanalytical method validation was performed in adherence to the FDA and ICH M10 guidelines^33,34^. This involved the assessment of parameters including selectivity, specificity, matrix effect, calibration curves, sensitivity, accuracy, precision, carry-over, recovery, and stability.

#### Selectivity

The method’s ability to selectively detect the analytes and IS without interference from the biological matrix was evaluated. Selectivity was evaluated using six independent lots of blank human plasma to investigate potential interferences at the retention times of the analytes and their respective IS. For mixture 1, the blank samples were evaluated against plasma spiked with DXM and PSE at their respective LLOQ of 0.05 ng/mL and 2.0 ng/mL, using OLA as the IS. For mixture 2, blanks were compared against plasma spiked with FLU and OLA at their respective LLOQ of 0.5 ng/mL and 0.2 ng/mL, using DXM as the IS. Each blank plasma sample underwent the full extraction protocol. In accordance with guidelines, the evaluation must demonstrate that no significant response attributable to interfering components is observed. Specifically, any responses from interfering components must not be more than 20% of the analyte response at the LLOQ, and not more than 5% of the IS response.

#### Matrix effect

For the matrix effect study, 6 different lots of plasma were investigated. For each lot, to prepare post-extraction spikes, blank human plasma samples were subjected to protein precipitation using 1 mL of acetonitrile.

For mixture 1, the resulting extracted supernatants were then spiked with DXM and PSE to achieve concentrations equivalent to the LQC (0.15 ng/mL for DXM; 6 ng/mL for PSE) and HQC concentrations (20 ng/mL for DXM; 800 ng/mL for PSE) for mixture 1. The IS (OLA), was added to a final concentration of 7500 ng/mL. The prepared samples were analyzed and compared against pure standard solutions of the same concentrations that were prepared directly in acetonitrile.

For mixture 2, the resulting extracted blank plasma supernatants were then spiked with OLA and FLU to achieve concentrations equivalent to the LQC (0.6 ng/mL for OLA; 1.5 ng/mL for FLU) and HQC concentrations (16 ng/mL for OLA; 40 ng/mL for FLU) along with DXM IS (500 ng/mL). Aliquots of these prepared samples were analyzed and compared with neat standard solutions of OLA and FLU prepared at equivalent concentrations directly in acetonitrile.

The matrix effect was evaluated by calculating the internal standard (IS)–normalized matrix factor (IS-MF). The IS-MF was obtained by dividing the analyte-to-IS peak area ratio in post-extraction spiked matrix samples by the corresponding ratio in neat solution (in absence of matrix). For the method to demonstrate reliable matrix effect performance, the coefficient of variation (CV%) of the IS-MF was required to be less than 15% across at least six individual matrix lots.

#### Calibration curves range and sensitivity

The selected calibration ranges were established based on reported plasma concentration levels (C_max_) following therapeutic dosing of the studied analytes following therapeutic dosing. This ensures the method provides comprehensive coverage of sub-therapeutic, therapeutic, and toxic concentrations.

For mixture 1, The calibration curves were constructed for DXM and PSE by plotting the "analyte response/IS response" peak area ratios against the corresponding nominal analyte concentrations. This was performed over a concentration range of 0.05–25.0 ng/mL for DXM and 2.0–1000.0 ng/mL for PSE.

For mixture 2, the calibration curves for OLA and FLU were constructed over a range of 0.2–20.0 ng/mL for OLA and 0.5–50.0 ng/mL for FLU by plotting the "analyte response/IS response" peak area ratios against the corresponding nominal analyte concentrations.

For all back-calculated concentrations on the calibration curves of mixture 1 and 2, the accuracy was assessed by confirming that deviations from the nominal value were within ± 15%. A broader deviation of up to ± 20% was considered acceptable for the LLOQ. The LLOQ was defined as the lowest concentration that could be quantified with acceptable accuracy and precision (± 20%), in accordance with FDA and ICH M10 bioanalytical method validation guidelines.

#### Accuracy and precision

The accuracy and precision of the method were evaluated using four QC levels (LLOQ, LQC, MQC, and HQC). Within-run (intra-day) accuracy and precision were evaluated by analyzing six replicates at each QC level in a single run, while between-run (inter-day) accuracy and precision were assessed by analyzing each QC level across three independent runs performed on three different days. Accuracy was simultaneously determined by calculating the (% nominal) from the comparison of the experimentally observed concentrations against their nominal values. Precision was assessed by determining the coefficient of variation (CV%). In accordance with bioanalytical guidelines, accuracy was required to be within ± 15% of the nominal concentration and precision within ± 15%, with limits extended to ± 20% at the LLOQ.

#### Recovery

The recovery of DXM, PSE, FLU and OLA was assessed by comparing the analytical responses of pre-extraction QC samples at low, medium, and high concentrations with those of post-extraction spiked samples. These post-extraction spikes were prepared by extracting blank plasma and then spiking it with the analytes at corresponding nominal QC levels. That was required to demonstrate reproducible recovery across all analyzed concentrations.

#### Carry-over

A carry-over assessment is vital for ensuring that there is no significant contamination carried over from previous high concentration runs that could compromise the integrity of subsequent analyses. This involved analyzing a blank plasma sample directly following the CS at the upper limit of quantification (ULOQ). The primary objective was to confirm that the carry-over from DXM, PSE, and the IS (OLA) remained below the accepted limits, as well as for OLA and FLU using DXM as IS. These limits are typically established as less than 20% of the LLOQ for analytes and less than 5% for the IS.

#### Stability

The stability of DXM, PSE, FLU and OLA were investigated at LQC and HQC concentrations under conditions simulating typical sample handling and storage. Short-term (bench-top) stability was assessed by keeping QC samples at room temperature for approximately 8 h, while long-term stability was determined after storage at − 20 °C for 30 days. Freeze–thaw stability was also evaluated by subjecting the QC samples to three complete cycles, with each cycle involving freezing at − 20 °C for 12 h followed by thawing at room temperature. Additionally, autosampler stability was evaluated after storage in the autosampler at 4 °C for 24 h. At each condition, stored QC samples were quantified using freshly prepared calibration standards and compared with freshly prepared QC samples at the corresponding concentration levels. The stability of the stock solutions for all analytes was assessed by comparing the analytical responses of solutions kept at 4 °C for 30 days with those of freshly prepared standards.

### Greenness assessment using AGREE and Auto-AGREE approach

To provide a quantitative and objective assessment of the method’s environmental impact, its greenness was evaluated using the Analytical GREEnness (AGREE) metric software^37^. To evaluate the capability of artificial intelligence in determining analytical greenness, Gemini Pro large language model (Google), was employed to execute an automated AGREE assessment (Auto-AGREE).

For prompt design and input structure, the foundational AGREE paper and its supplementary data^37^ were uploaded directly to the AI model, alongside the proposed LC–MS/MS analytical procedure. To ensure correct application of the evaluation criteria, the prompt instructed the model to extract the relevant analytical parameters and mathematically calculate the scores for the 12 AGREE principles according to the established framework. For reproducibility check, the same input conditions were maintained across repeated runs.

For validation of the AI-generated outputs, including the extracted input parameters, individual principle scores, overall composite score, color assignments, and the corresponding pictogram, ere systematically cross-checked by the authors against the results produced by the official AGREE software to confirm agreement.

### Whiteness assessment using RGB12 and Auto-RGB12 approach

The proposed method’s analytical performance, greenness, and operational efficiency were evaluated using the White Analytical Chemistry (WAC) approach based on the RGB12 model^38^. To evaluate the capability of artificial intelligence in performing whiteness assessment, Gemini Pro large language model (Google), was employed to execute an automated RGB12 assessment (Auto-RGB12).

For prompt design and to ensure the model applied the correct evaluation criteria, the foundational WAC reference paper and its supplementary data^38^ were uploaded to the AI, alongside the developed method’s experimental procedures and final analytical results. The AI was tasked with extracting the relevant method parameters and mathematically calculating the scores for the 12 WAC principles of the RGB model and the final white score. Repeated evaluations under identical input conditions produced for variability checks.

To validate AI-generated results, the 12 principles score calculations, the resulting overall white score, and the chart generated by Gemini Pro were cross-checked by the authors against the results produced by the official RGB12 software to confirm agreement.

## Results and discussion

The binary mixture of DXM and PSE are provided in a fixed dose formulation for cough/cold treatment, while the binary mixture of OLA and FLU are provided in a fixed dose formulation for the treatment of depression. Both formulas are subjected to abuse for their euphoric effect in high doses. In the ongoing effort to precisely and efficiently analyze drug combinations within biological samples, LC–MS/MS has become the gold standard. Its key advantages, including high sensitivity, rapid throughput, and exceptional specificity, make it a valuable tool for thorough pharmacokinetic investigations, therapeutic drug monitoring and toxicological analysis.

### Method development and optimization

The use of acetonitrile-induced protein precipitation was selected as a simple, rapid, and universally applicable extraction approach for the simultaneous analysis of structurally diverse analytes in human plasma. This technique offers efficient protein removal with minimal sample handling and is fully compatible with LC–MS/MS analysis. The 1:1 ratio resulted in incomplete precipitation and cloudy supernatants. A ratio of 1:2 (plasma: acetonitrile, v/v) was selected because it provided clear supernatant formation, and consistent extraction performance for all analytes. Increasing the acetonitrile volume beyond this ratio did not result in any significant improvement in recovery, matrix effect, or chromatographic response and would unnecessarily increase solvent consumption. The effectiveness of the extraction procedure was confirmed by high and consistent recoveries, negligible matrix effects, and good reproducibility. The results demonstrated that the internal standard–normalized matrix factor (IS-MF) showed low variability (CV ≤ 15%) across different plasma lots (Table 1), confirming negligible matrix effects and good reproducibility. In addition, the consistent recovery (> 90%) across QC levels supports the reliability of the extraction procedure (Table S3). These findings collectively confirm that the method effectively compensates for potential variability arising from differences in plasma matrix composition and analyte physicochemical properties. Although alternative extraction approaches such as SPE and LLE may provide high selectivity, the selected protein precipitation procedure offered an optimal balance between simplicity, throughput, recovery, and matrix effect performance for the intended routine bioanalytical application.

**Table 1:** ME study results at LLOQ and HQC levels across 6 plasma lots.

	DXM		PSE		OLA		FLU	
	LOQ	HQC	LOQ	HQC	LOQ	HQC	LOQ	HQC
Mean of IS-MF ratio	1.01	1.05	1.23	1.15	1.19	0.99	1.20	0.99
CV%	5.941	0.952	7.317	3.47	5.88	4.04	8.33	1.01

Although different internal standards were employed for each binary mixture, the overall analytical platform remains unified, as both mixtures were analyzed under identical sample preparation procedure, chromatographic conditions, and mass spectrometric operating conditions. The selection of internal standards was guided by analytical suitability to ensure optimal correction of matrix effects and ionization variability within each analyte group. Therefore, the approach maintains a unified analytical platform while applying internally optimized correction strategies to ensure quantitative reliability across structurally diverse compounds.

The rationale for utilizing a cross-internal standard strategy—OLA for Mixture 1 (DXM/PSE) and DXM for Mixture 2 (OLA/FLU)— was based on their demonstrated empirical suitability under the optimized LC–MS/MS conditions rather than structural similarity alone. Under the final chromatographic conditions, all analytes and their corresponding internal standards exhibited closely aligned retention times, resulting in a narrow retention window. This allowed near-simultaneous entry into the ESI source, exposing both analyte and internal standard to highly comparable ionization conditions and matrix environments. Consequently, the selected internal standards effectively compensated for localized matrix-induced ion suppression/enhancement, as confirmed by acceptable matrix effect results (IS-normalized matrix factors close to unity with %CV < 15%), as well as satisfactory accuracy and precision across all QC levels. This cross-internal standard approach, combined with the shared basic character and strong positive ESI response of all compounds, provided reliable quantitative performance for routine bioanalytical application.

Several chromatographic parameters were sequentially optimized to achieve efficient separation, symmetrical peak shape, adequate sensitivity, and satisfactory resolution for the target analytes. During preliminary method development, initial trials using a C8 stationary phase resulted in poor analyte retention and suboptimal peak shapes. Therefore, a more retentive Hypersil Gold C18 column (100 mm × 3 mm, 1.9 μm) was selected. The 1.9 μm particle size classifies the method within the domain of ultra-high-performance liquid chromatography (UHPLC), significantly increasing column efficiency. This leads to the sharper peaks and enhanced resolution critical for separating analytes from the complex plasma matrix. Furthermore, the use of a narrow-bore column (3.0 mm ID) is a key feature of modern UHPLC, allowing for a dramatic reduction in mobile phase consumption (0.75 mL per run) while maintaining excellent chromatographic performance.

The mobile phase composition was systematically optimized to achieve distinct peaks and high sensitivity at the LLOQ. Initial trials using methanol as the organic modifier resulted in poor peak shape and noticeable tailing. Consequently, acetonitrile was selected due to its lower viscosity and superior elution strength, which significantly improved peak symmetry. Various ratios of aqueous to organic phases were evaluated; increasing the aqueous proportion led to undesirable peak broadening. Optimal chromatographic performance was ultimately achieved using an isocratic elution of 0.1% formic acid and acetonitrile (30:70, v/v). The flow rate was set to 0.3 mL/min, with the column temperature maintained at 30 °C. The entire chromatographic separation was completed in a rapid runtime of 2.5 min enabling high-throughput analysis.

Positive electrospray ionization (ESI+) mode was utilized, yielding abundant [M + H]^+^ precursor ions for all analytes. The optimized multiple reaction monitoring (MRM) transitions, collision energies, and final source parameters are summarized in Table S1. Fragmentation produced characteristic product ions aligned with established literature. PSE yields an abundant *m/z* 148.1 ion via the loss of water (18 Da) from its hydroxyl group^39–41^. DXM generates a dominant fragment at *m/z* 215.1 via cleavage of the morphinan nitrogen-containing bridge, producing a stable tricyclic ion^42,43^. OLA produces a major product ion of *m/z* 256.05 via charge-directed α-cleavage at the at the piperazine moiety, involving the loss of a 57 Da neutral fragment^44–46^. Finally, FLU fragmented predominantly to m/z 148.1 due to ether bond cleavage forming a stable phenylpropylamine ion^46,47^.

A key operational advantage of the proposed LC–MS/MS method is its ability to determine two pharmacologically distinct binary mixtures (DXM/PSE and OLA/FLU) within a single analytical sequence under identical chromatographic and MS/MS conditions. While pharmacologically distinct, both pairs are associated with significant abuse potential and are frequently encountered in misuse-related contexts. This unified applicability to both mixtures is supported by their compatible analytical behavior, including efficient chromatographic separation under a single mobile phase system, effective ionization in positive ESI mode, and suitability for a common protein precipitation sample preparation protocol. From an operational perspective, this unified approach eliminates the need for method switching, thereby reducing instrument downtime and streamlining analytical workflows in clinical and forensic settings.

Notably, dedicated LC–MS/MS methods for the simultaneous determination of DXM and PSE in human plasma remain scarcely reported, despite their frequent co-administration and abuse potential, representing a significant bioanalytical gap in the literature.

In parallel, the proposed method was evaluated against previously reported LC–MS/MS methods for OLA and FLU (Table S2). The proposed method demonstrates substantial advantages in terms of simplicity of sample preparation, cost-effectiveness, and analytical throughput. A major limitation of previously reported methods is their reliance on complex, labor-intensive, or costly sample preparation procedures. Methods employing solid-phase extraction^29,31^, although highly selective, require expensive disposable cartridges and involve multi-step processing. Similarly, the method reported by Fan et al.^29^utilizes an online SPE configuration which, despite being automated, requires a specialized and costly multi-pump, valve-switching LC system. Conversely, methods based on liquid–liquid extraction (LLE)^28,32^ require the additional evaporation and reconstitution steps, and may suffer from poor extraction efficiency. For example, Ni et al.^28^ reported average recoveries of approximately 60% for FLU using LLE. In contrast, the present method employs a rapid, single-step protein precipitation procedure, thereby maximizing throughput while minimizing sample handling and potential analyte loss. Furthermore, some previously reported methods rely on expensive stable isotope-labeled internal standards to compensate for extraction variability and matrix effects^28,30^. In contrast, the proposed assay achieves acceptable and reproducible precision and effective matrix effect correction using readily available and cost-effective internal standards. The proposed method also demonstrates improved chromatographic performance through the use of a sub-2-µm UHPLC column (1.9 μm particle size, 3.0 mm internal diameter), which enables efficient separation with symmetrical peak shapes within a short total runtime of 2.5 min. In comparison, previously reported methods predominantly employed conventional 5.0 μm, 4.6 mm i.d. columns, requiring high flow rates of up to 1.0 mL/min^28,29^ and significantly longer analysis times, reaching up to 11 min^29^. By optimizing the chromatographic conditions, the present method reduces organic solvent consumption to 0.75 mL per run, representing a decrease of approximately 41.4–93.2% relative to conventional high-flow methods. This reduction in mobile phase consumption minimizes hazardous waste generation and lowers operational costs while supporting the principles of green analytical chemistry (GAC).

### Method validation

#### Selectivity

**Fig. 2:**
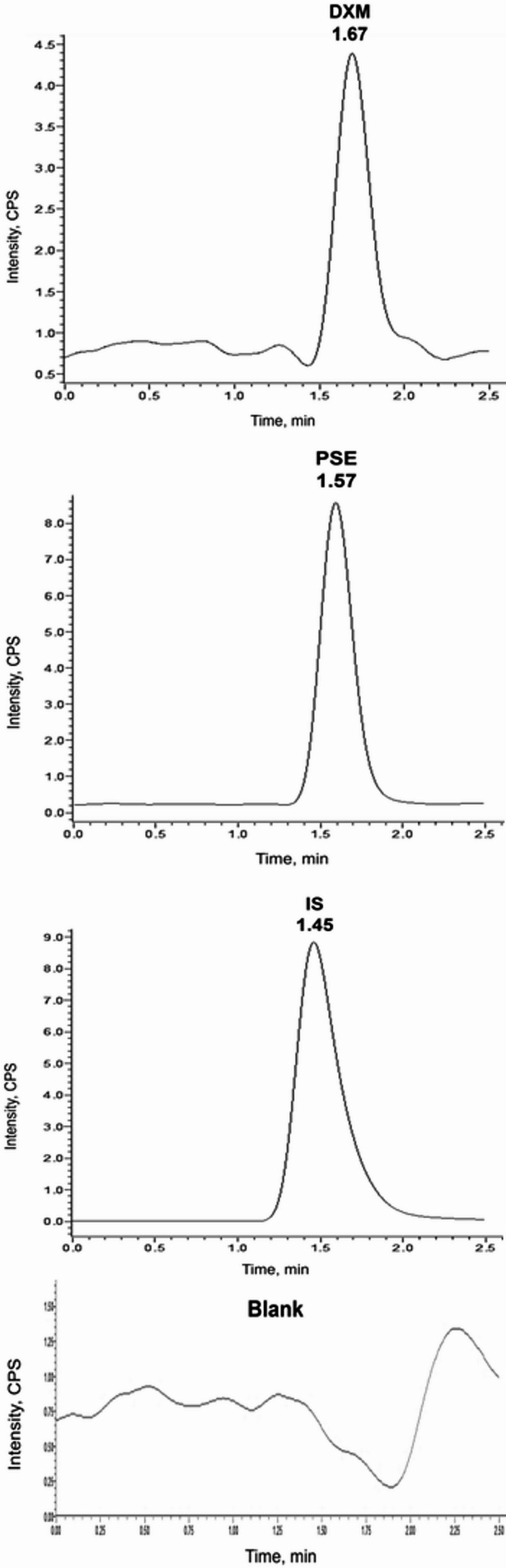
Representative MRM chromatograms of: DXM 0.05 ng/mL (LLOQ), PSE 2.0 ng/mL (LLOQ), OLA (IS) for mixture 1, and blank human plasma.

**Fig. 3:**
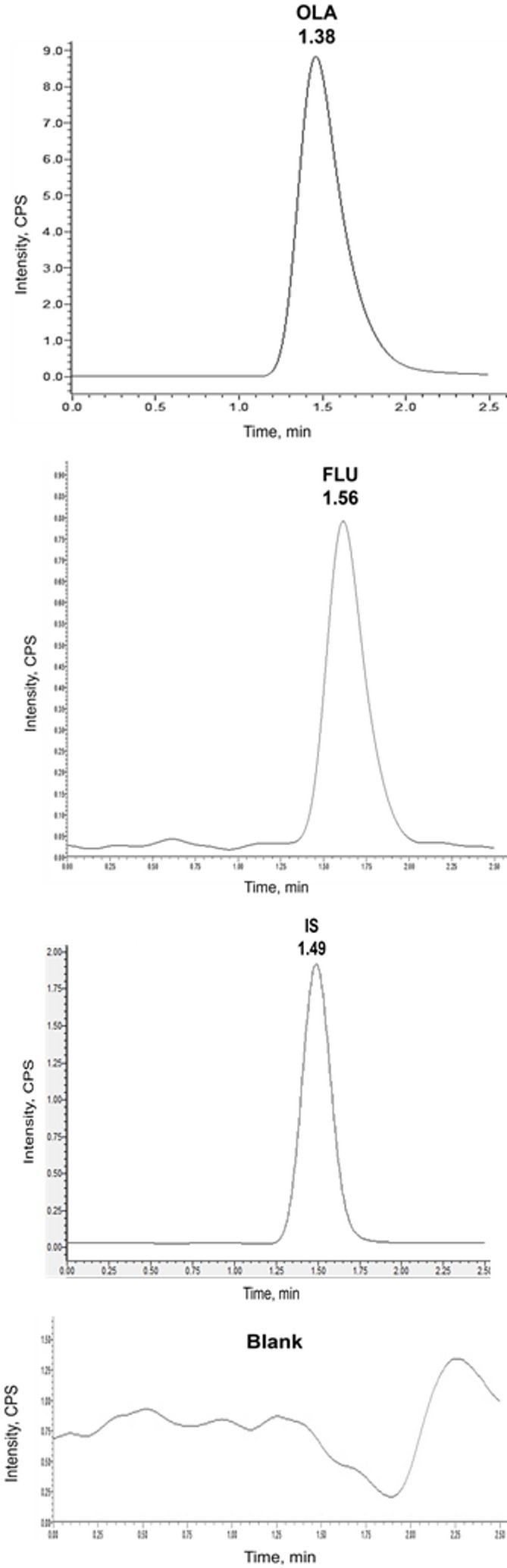
OLA 0.2 ng/mL (LLOQ), FLU 0.5 ng/mL (LLOQ), DXM (IS) for mixture 2, and blank human plasma.

Selectivity was confirmed by the analysis of the blank plasma extracts, which showed the absence of any significant endogenous interferences at the retention times of the analytes (DXM, PSE, OLA, FLU) and their respective internal standards. This was verified by directly comparing the blank chromatograms with those obtained from plasma spiked at the lower limit of quantification (LLOQ). The results demonstrate that the method is highly selective and free from matrix interferences, ensuring the accurate and reliable quantification of all target analytes. Representative chromatograms illustrating this selectivity are presented in Figs. 2 and 3.

#### Matrix effect

To ensure the reliable and accurate quantification of DXM, PSE, FLU, and OLA in human plasma using the proposed method, the potential for matrix effects, a phenomenon where co-eluting plasma components can interfere with the ionization of the analytes, was thoroughly assessed using the Internal Standard (IS) Normalized Matrix Factor (IS-MF). Matrix effect evaluation was performed using six different plasma sources at both LQC and HQC concentration levels through assessment of internal standard–normalized matrix factors.

As summarized in Table 1, the coefficient of variation (CV%) of the IS-MF values remained within the regulatory acceptance limit of ≤ 15% for LQC and HQC of all analytes, demonstrating acceptable reproducibility across the evaluated plasma lots. Although certain analytes exhibited IS-MF values greater than unity, indicating limited ion enhancement, these effects were consistent and compensated by the selected internal standards. Collectively, these results suggest that the optimized sample preparation conditions and reliable performance of the ISs of the internal standards contribute to minimizing ion suppression or enhancement effects, supporting reliable quantification.

#### Calibration curves range and sensitivity

A linear relationship was observed between analyte-to-internal standard peak area ratios and analyte concentrations using unweighted least-squares regression.

For Mixture 1 (DXM/PSE), calibration curves constructed in spiked human plasma demonstrated excellent linearity over the concentration ranges of 0.05–25.0 ng/mL for DXM and 2.0–1000.0 ng/mL for PSE. The corresponding regression equations:

For DXM: *y* = 0.0014*x* + 0.001, (*r*^*2*^ = 0.9992)

For PSE: *y* = 0.0031*x* + 0.0307, (*r*^*2*^ = 0.9999)

The LLOQs were established at 0.05 ng/mL and 2.0 ng/mL for DXM and PSE, respectively.

Similarly, Mixture 2 (OLA/FLU) exhibited consistent linearity over 0.2–20.0 ng/mL for OLA and 0.5–50.0 ng/mL for FLU, with regression equations of:

For OLA: *y* = 0.777*x* + 0.0652, (*r*^*2*^ = 0.9996)

For FLU: *y* = 0.8604*x* + 0.7812, (*r*^*2*^ = 0.999)

The LLOQs were 0.2 ng/mL for OLA and 0.5 ng/mL for FLU.

All back-calculated concentrations for the CSs were within ± 15% of their nominal values, except for the LLOQ, where a deviation of up to ± 20% was considered acceptable. The selected LLOQ values demonstrated acceptable precision, accuracy, selectivity, and signal response.

#### Accuracy and precision

The developed LC–MS/MS method for the drugs under investigation in human plasma underwent a thorough assessment of accuracy and precision, through both intra-day and inter-day studies, in compliance with established guidelines. Both within-run (intra-day) and between-run (inter-day) studies were performed, with predefined acceptance criteria of ± 15% for all QC levels, and within ± 20% for the LLOQ. Detailed results for within-run and between-run accuracy and precision are presented in Table 2.

**Table 2:** Intra- and inter-day precision and accuracy results.

Drugs	QC Level	Within-run (n = 6)		Between-run (n = 3 × 6)	
		Mean accuracy (%)	Precision (CV %)	Mean accuracy (%)	Precision (CV %)
DXM	LLOQ	95.10	14.406	90.40	8.799
	LQC	102.38	7.306	97.75	5.082
	MQC	108.75	4.855	105.65	2.408
	HQC	105.16	0.580	105.45	1.384
PSE	LLOQ	84.03	1.916	84.00	6.320
	LQC	92.03	5.248	91.29	5.071
	MQC	102.19	1.272	103.64	2.032
	HQC	94.04	1.755	96.13	1.776
OLA	LLOQ	80.00	0.313	82.93	1.729
	LQC	97.57	0.625	103.07	6.241
	MQC	108.39	1.891	105.24	2.665
	HQC	96.28	1.163	98.14	3.639
FLU	LLOQ	86.36	3.381	84.19	4.238
	LQC	91.71	3.805	91.69	3.622
	MQC	104.54	9.040	104.74	5.578
	HQC	91.12	1.635	92.10	1.477

*Accuracy values represent the mean % of nominal concentration obtained from replicate QC analyses.

All within-run and between-run accuracy and precision results for the four analytes met the predefined acceptance criteria, demonstrating acceptable and reproducible analytical performance across the validated concentration ranges. Although the within-run accuracy for OLA at the LLOQ was at the lower acceptance boundary (80.0%), it remained fully compliant with ICH M10 and FDA bioanalytical method validation guidelines, which permit deviations within ± 20% at the LLOQ. The corresponding precision was excellent (CV = 0.31%), confirming reliable quantification at the lower end of the validated concentration range.

#### Recovery

The extraction recovery of DXM, PSE, and the IS (OLA) for mixture 1 or OLA and FLU with the IS (DXM) for mixture 2 from human plasma was evaluated to confirm efficiency of the protein precipitation method. This was determined by comparing the analytical responses of analytes in extracted QC samples (low, medium, and high concentrations) with those from corresponding post-extracted standards. The recovery results at LQC, MQC, and HQC levels are summarized in Table S3. The results confirmed good and consistent extraction recoveries across the tested concentrations, showing that the protein precipitation procedure provided reliable extraction efficiency.

#### Carry-over

A carry-over assessment was conducted to ensure the absence of residual analytes or IS from previous runs, which is essential for maintaining the accuracy of subsequent measurements. Blank plasma samples were injected immediately after the analysis of the ULOQ samples. To quantify the carry-over, the peak areas in blank samples injected after the ULOQ were compared to the LLOQ responses. The observed carry-over was found to be minimal, with maximum observed values of 0.61% for DXM, 0.83% for PSE, 0.52% for OLA, and 2.2% for FLU; all were significantly below the regulatory limit of ≤ 20% of the LLOQ response. For the internal standards, carry-over did not exceed 1.9%, remaining well within the acceptance criterion of ≤ 5% of the mean IS response. These results confirm negligible carry-over under the optimized analytical conditions.

#### Stability

The stability of DXM, PSE, OLA, and FLU in human plasma was evaluated under different storage and handling conditions, including short-term, long-term, freeze–thaw, and autosampler stability. The measured concentrations at LQC and HQC levels remained within ± 15% of freshly prepared samples, with precision (CV%) ≤ 15% across all conditions (Table 3), indicating acceptable stability under the tested conditions with no significant loss of analyte concentration observed.

**Table 3:** Stability of DXM, PSE, OLA, and FLU under various storage conditions.

Storage Conditions	QC level	DXM		PSE		OLA		FLU	
		% Of nominal concentration	Precision (CV %)	% Of nominal concentration	Precision (CV %)	% Of nominal concentration	Precision (CV %)	% Of nominal concentration	Precision (CV %)
Short-term (Bench-top) (8 h, room temperature)	LQC	99.23	2.063	98.94	3.621	105.23	3.062	99.46	2.652
	HQC	102.64	2.713	102.75	4.223	101.67	2.931	96.71	2.332
Long-term (30 days, − 20 °C)	LQC	95.49	3.442	96.15	2.379	98.65	4.553	101.53	3.744
	HQC	98.16	4.326	101.36	3.98	97.94	4.22	98.23	5.23
Freeze–thaw (3 cycles)	LQC	93.75	1.549	97.35	1.954	94.57	2.086	102.2	2.54
	HQC	102.63	2.91	103.42	2.58	99.63	3.325	101.35	4.771
Autosampler (24 h, 4 °C)	LQC	101.57	2.081	96.94	1.971	102.16	1.486	96.18	2.551
	HQC	103.82	4.068	98.83	3.218	103.37	3.651	99.87	2.741

*n = 6 per QC level.

In addition, the stability of stock and working standard solutions was evaluated at room temperature for 8 h (short-term) and after refrigerated storage at 4 °C for 30 days (long-term). DXM, PSE, FLU and OLA stock and working solutions showed no evidence of degradation under these conditions, with measured responses remaining within ± 2% of freshly prepared solutions. This confirms that stock solutions can be reliably used for up to one month when refrigerated, facilitating high-throughput routine analysis.

### System suitability

System suitability was verified prior to the start of each analytical batch through six consecutive injections (n = 6) of a mid-level quality control (MQC) sample. The results demonstrated excellent repeatability, with the coefficient variation (CV%) of peak area ratios and retention times remaining within acceptable limits (≤ 2%) for all analytes and internal standards as presented in Table S4.

### Evaluation of the greenness and whiteness of proposed methods

While the AGREE metric specifically evaluates the environmental greenness of the analytical procedure according to the 12 principles of Green Analytical Chemistry, the RGB12 model provides a more comprehensive assessment by integrating analytical performance, environmental impact, and practical efficiency within the White Analytical Chemistry framework.

#### Application of AGREE metric for greenness assessment

The environmental impact of analytical procedures has become an important consideration in method development. It should be noted that LC–MS/MS methods involving biological matrices inherently present environmental limitations due to sample preparation requirements and solvent consumption. In this context, the present study aimed to develop an analytical method that is relatively greener compared to conventional LC–MS/MS methods, while maintaining high analytical performance. Conventional analytical techniques often require large volumes of organic solvents and generate considerable waste; therefore, efforts were directed toward reducing solvent consumption, minimizing sample preparation steps, and lowering energy demand. The method employs a short 2.5-min runtime to reduce instrument energy consumption, solvent usage (≈0.75 mL mobile phase per injection), and waste generation. The use of 0.1% formic acid, a volatile and MS-compatible modifier, further limits hazardous waste. In addition, a simple protein precipitation procedure was adopted to minimize sample handling steps and reduce the volume of organic precipitating solvent compared with more complex extraction techniques. Although acetonitrile consumption during the protein precipitation step contributes to the overall environmental impact of the method, the selected extraction procedure remains operationally simpler and generally requires fewer processing steps compared with conventional liquid–liquid extraction and solid-phase extraction approaches. Furthermore, the development of a unified method for the simultaneous determination of the studied analytes provides practical advantages for clinical and forensic laboratories, improving workflow efficiency and reducing instrument downtime associated with multiple analytical methods. In addition, the use of a single analytical platform reduces overall resource consumption compared to multiple independent methods, further supporting its practical and economic advantages.

**Fig. 4:**
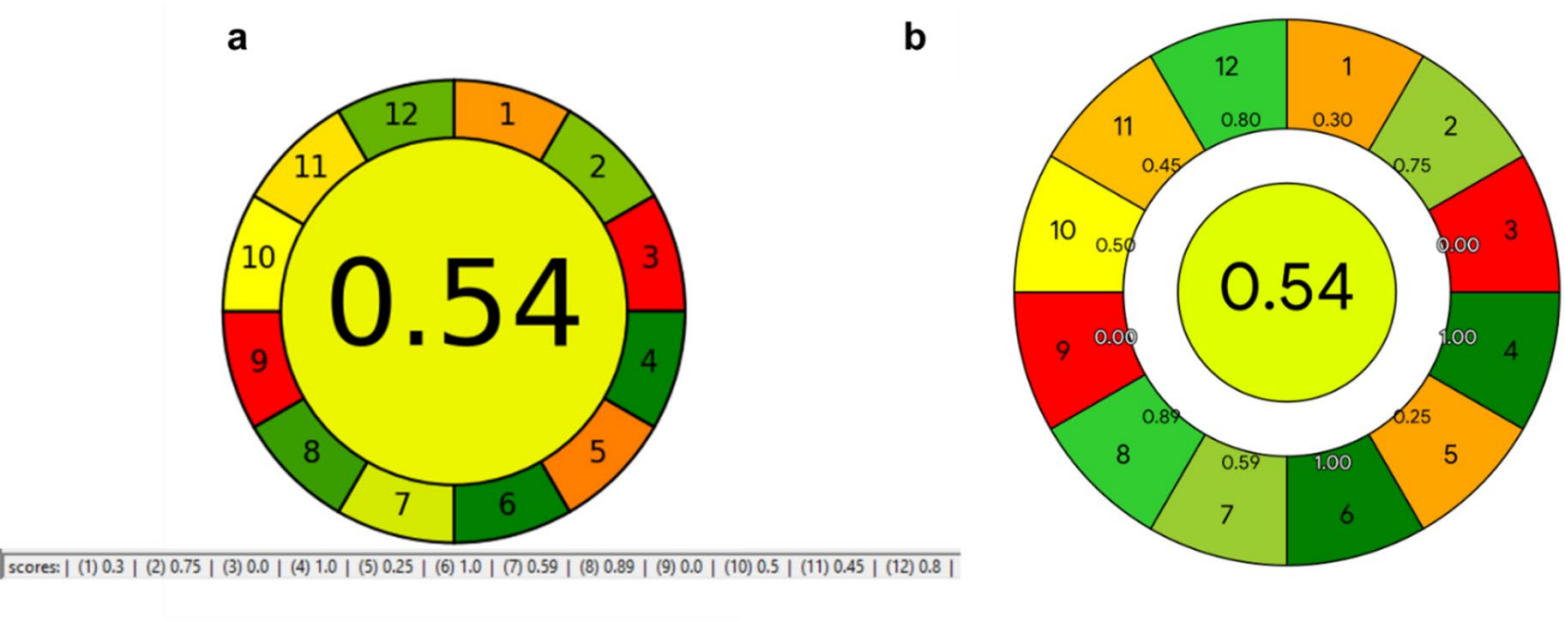
Greenness assessment of the proposed LC–MS/MS method according to: (**a**) The AGREE metric (manual assessment), and (**b**) Auto-AGREE (AI-assisted assessment using Gemini Pro).

To provide a quantitative and objective assessment of the method’s environmental impact, its greenness was evaluated using the Analytical GREEnness (AGREE) metric software^37^, which is based on the 12 Principles of Green Analytical Chemistry (GAC). The output is presented as a clock-like graph, with the overall score displayed at the center along with a corresponding color representation. The Green indicates good compliance (≥ 0.7), Yellow indicates moderate compliance (0.4–0.69), and Red indicates weak compliance (< 0.4). The AGREE pictogram for the proposed method, with an overall greenness score of 0.54, is presented in Fig. 4a.

#### Application of RGB model for whiteness assessment

**Fig. 5:**
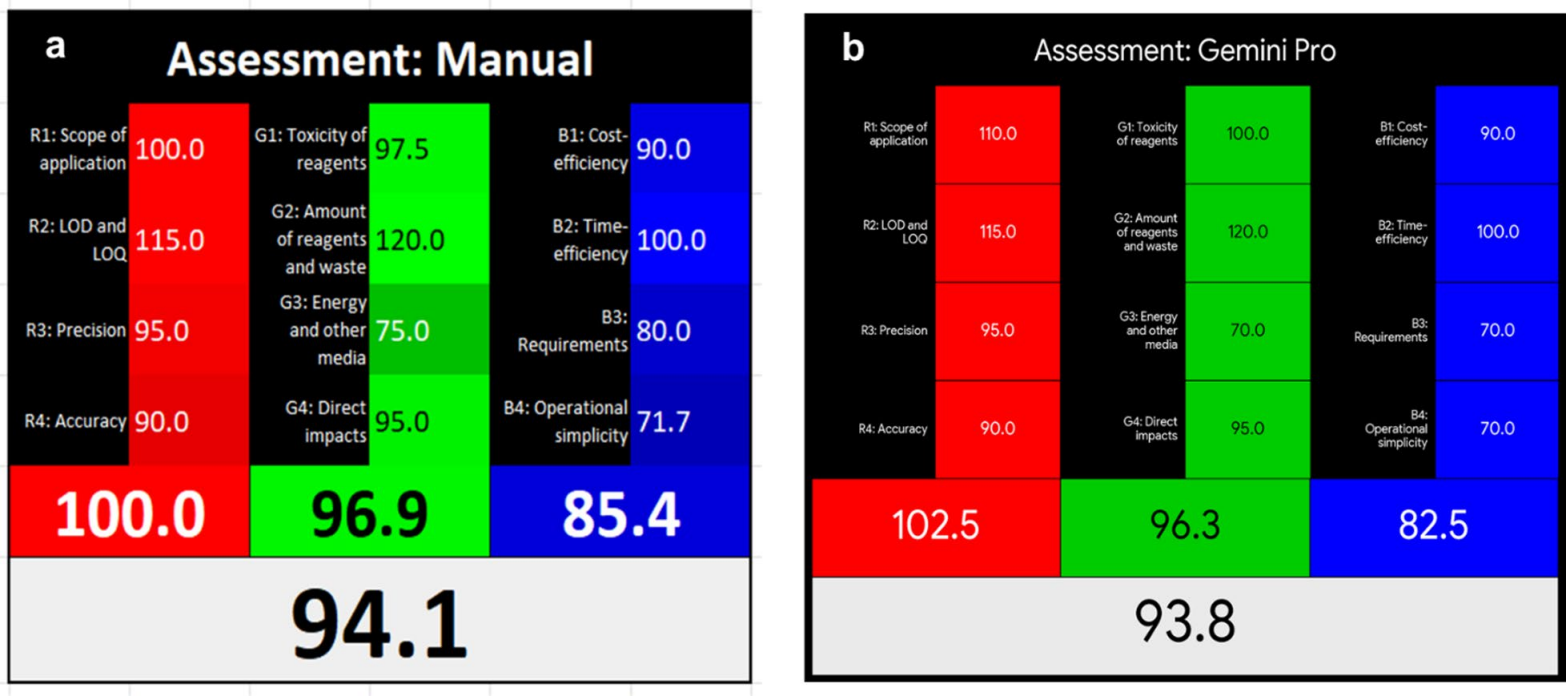
Whiteness assessment of the proposed LC–MS/MS method according to: (**a**) The RGB 12 model (manual assessment), and (**b**) Auto-RGB 12 (AI-assisted assessment using Gemini Pro).

White Analytical Chemistry (WAC)^38^, is a newly introduced concept for evaluating analytical methods based on the synergy between analytical performance, environmental impact, and practical efficiency. The concept is inspired by the color white, which results from combining the primary colors red, green, and blue (RGB). In the WAC approach, the RGB models are utilized to comprehensively assess analytical methods across three dimensions. The red model evaluates the effectiveness of analytical method through four key principles: scope of application (R1), LOD and LOQ (R2), precision (R3), and accuracy (R4). The green model considers the four fundamental GAC principles for measuring the greenness of analytical procedures, focusing on the toxicity of reagents (G1) and the reagents consumption and waste production (G2). It also accounts for energy consumption (G3), and direct impacts (G4), which evaluates occupational safety and hazards to the analyst. The blue model assesses practicality and operational efficiency, considering cost-effectiveness (B1), time efficiency (B2), sample consumption and other advanced requirements (B3), and operational simplicity (B4). The proposed LC–MS/MS was evaluated using the RGB12 model, with a score of 94.1% (Fig. 5a). This indicates an optimal balance of holistic sustainability, where high analytical performance (Red) is achieved without compromising its environmental performance relative to conventional LC–MS/MS methods (Green) or practical economic applicability (Blue). Unlike methods that prioritize one aspect at the expense of others, this high score confirms the method is a reliable, eco-friendly, and cost-effective tool. The higher RGB12 score relative to the AGREE score reflects the broader scope of the RGB12 model, which evaluates not only environmental impact but also analytical performance and practical efficiency within the White Analytical Chemistry framework.

#### Application of AI models (Auto-AGREE and Auto-RGB 12) for AI-Assisted greenness and whiteness assessments

Recently, artificial intelligence (AI) models have been piloted in sustainability assessment, demonstrating their potential in objective evaluation^48^. In this study, AI was not employed for analytical method development or optimization, but rather as a supportive tool for the automated application of established sustainability assessment frameworks.

The AI model (Google Gemini Pro) was not used to generate or infer the assessment criteria; rather, it was used solely to automate the calculation and visualization of scores according to the established AGREE and RGB12 frameworks. The foundational AGREE publication and its supplementary materials, as well as the White Analytical Chemistry (WAC) reference paper and its supplementary materials, were uploaded directly to the AI model (Gemini Pro) together with the proposed LC–MS/MS analytical procedure and final analytical results. The prompts instructed the model to extract the relevant analytical parameters and mathematically calculate the scores for the 12 AGREE principles and the 12 WAC principles of the RGB12 model, including the final composite greenness and whiteness scores and their corresponding pictograms. To validate the results, the AI-generated outputs were cross-checked through a detailed point-by-point comparison against manually calculated AGREE and RGB12 assessments.

The comparison between the AI-assisted Auto-AGREE (Gemini Pro) approach and the conventional AGREE metric showed high agreement in the final score, individual principle evaluations, and color assessment producing a similar pictogram as shown in Fig. 4b, while the detailed AI-generated AGREE outputs, including principle-wise scores and color scale assignments, are provided in Table S5.

Similarly, the comparison between the AI-assisted Auto-RGB12 (Gemini Pro) approach and the conventional RGB12 model showed close agreement, yielding a whiteness score of 93.8% (Fig. 5b).

To assess output consistency and guard against AI hallucinations, the prompt sequence was run in triplicate. The AI-generated outputs were found to be consistent across repeated runs using the same structured input, indicating stable performance for the defined task.

This comparison indicates that universally accessible AI models such as Gemini Pro, can support structured and standardized sustainability assessment with reduced operator-dependent variability. By transitioning from manual scoring to AI-assisted workflow, the time and effort required for method evaluation can be significantly reduced while maintaining a standardized evaluation of analytical performance, environmental greenness, and practical applicability. Ultimately, these widely available AI models may support analytical chemists in evaluating the holistic sustainability of their methods in a more structured and transparent manner.

It should be noted that the performance of AI-assisted evaluation is dependent on the quality and structure of the input data, and the predefined assessment frameworks; therefore, expert oversight remains essential.

## Conclusion

This work addresses a unified LC–MS/MS method capable of determining DXM/PSE and OLA/FLU binary mixtures frequently associated with misuse and abuse. In addition, it addresses the limited availability of dedicated LC–MS/MS methods for the simultaneous quantification of DXM/PSE in human plasma, extending analytical capabilities beyond previously reported pharmaceutical or single-analyte approaches. The validated method demonstrated acceptable accuracy, precision, selectivity, and stability, with high sensitivity reflected by low LLOQ values of 0.05 ng/mL for DXM, 2.0 ng/mL for PSE, 0.2 ng/mL for OLA, and 0.5 ng/mL for FLU. Combining high sensitivity with operational efficiency, including a short 2.5-min runtime, reduced solvent consumption, and a simple protein precipitation procedure, the method represents a practical and reliable analytical platform with potential applicability to clinical, and forensic investigations. The unified approach provides operational and economic advantages over multiple separate assays, particularly in high-throughput laboratory settings.

Moreover, the AI model (Gemini Pro) was utilized to evaluate method greenness and whiteness using Auto-AGREE and Auto-RGB 12 tools. The AI model was utilized as a supportive tool to automate the application of established greenness and whiteness assessment metrics, and not for method development or optimization. The AI-generated results were systematically compared with those obtained through conventional manual input into the standard AGREE and RGB12 metrics. The comparative analysis demonstrated strong agreement between AI-assisted and conventional manual assessment approaches under the applied evaluation conditions, with highly comparable overall scores and evaluation outcomes. The transition from manual data entry to automated comparative analysis reduces operator-dependent variability and substantially reduces the time and effort required for comprehensive greenness and whiteness assessments. In addition, the AI model provides transparent reasoning for its selections and calculations, allowing analysts to verify and critically appraise the evaluation process. Nevertheless, despite the efficiency and consistency offered by AI-based tools, human expertise remains essential for accurate data input, and final validation of the assessments.

## Supplementary Information

Supplementary Information.

## Data Availability

The datasets used and/or analysed during the current study are available from the corresponding author on reasonable request.
