# Supplementary material for: AI-assisted versus manual sustainability assessment of a high-throughput LC–MS/MS method for psychotropic and OTC drugs of abuse in human plasma
Source: Sci Rep. 2026 Jul 6;16:20807. doi: 10.1038/s41598-026-59794-z (PMC13338246; doi:10.1038/s41598-026-59794-z)
Supplement: Supplementary file 1 — Supplementary Information. [file 41598_2026_59794_MOESM1_ESM.docx]

**Supplementary material**

**AI-assisted versus manual sustainability assessment of a high-throughput LC–MS/MS method for psychotropic and OTC drugs of abuse in human plasma**

Hend Z. Yamani^1^*, Khaled Hesham^2^,Shereen M. Tawakkol^2,^, Lobna A. Hussein^1^, Nesma M. Fahmy^2^

*^1^Pharmaceutical Analytical Chemistry Department, Faculty of Pharmacy, Ain Shams University, Cairo 11566, Egypt*

*^2^Pharmaceutical Chemistry Department, Faculty of Pharmacy, Ahram Canadian University, Giza, Egypt*

*Corresponding author e-mail: [hend.z.yamani@pharma.asu.edu.eg](mailto:hend.z.yamani@pharma.asu.edu.eg)

**
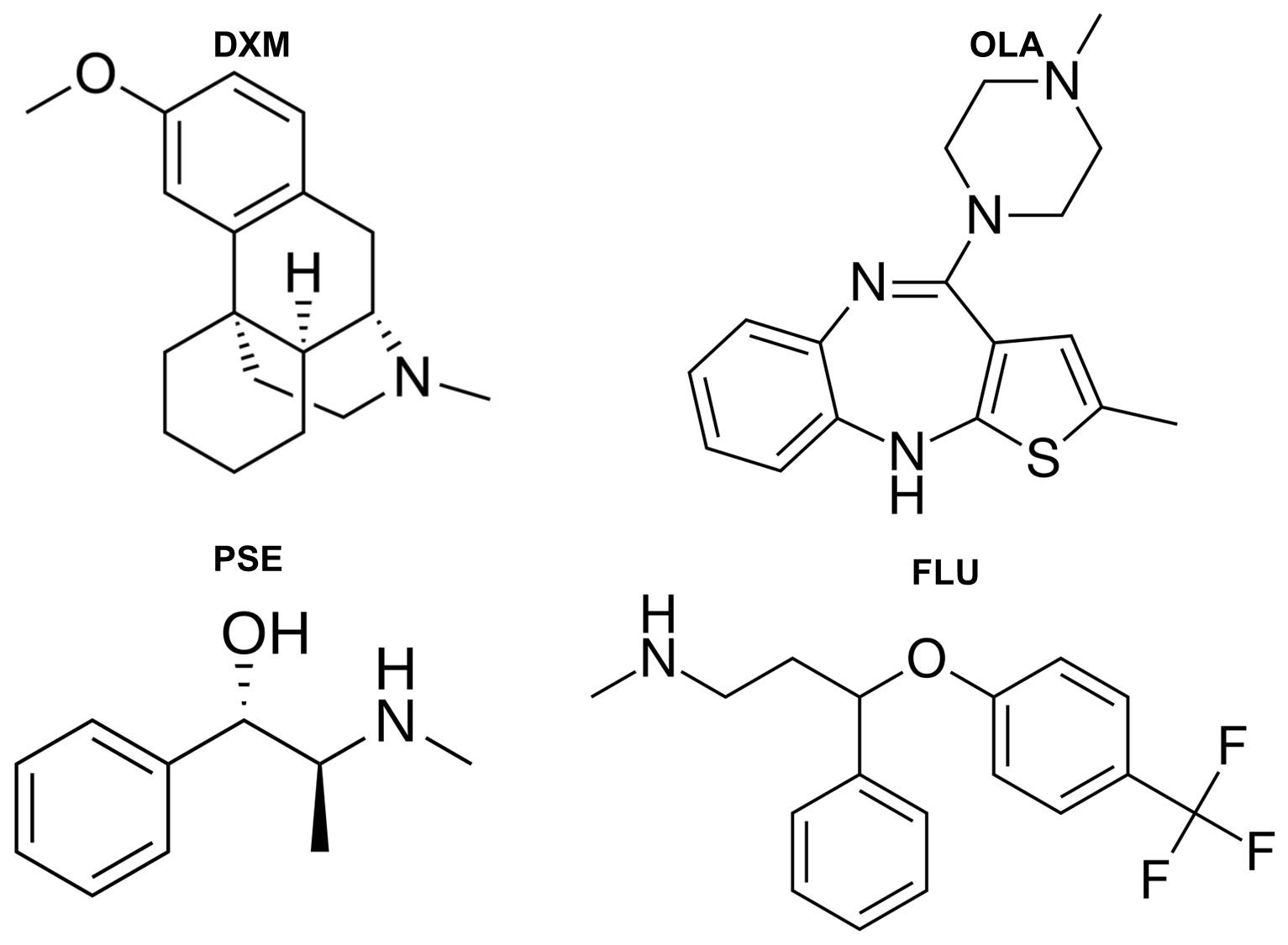
**

**Figure S1**: The chemical structure of DXM, PSE, OLA, and FLU

**Table S1:** Optimized LC-MS/MS Parameters for DXM, PSE, FLU and OLA.

| **Analyte** | **Q1 (m/ z)** | **Q3 (m/ z)** | **Collision Energy (V)** | **Dwell Time (ms)** |
| --- | --- | --- | --- | --- |
| DXM | 271.90 | 215.10 | -12 | 100 |
| PSE | 166.25 | 148.10 | -13 | 100 |
| OLA | 312.95 | 256.05 | -10 | 100 |
| FLU | 309.85 | 148.10 | -9 | 100 |
| Nebulizing gas flow | 3 L/min | | | |
| Drying gas flow | 15 L/min | | | |
| DL temperature | 250°C | | | |
| Heat block temperature | 400°C | | | |
| Interface voltage | 4.5 kV | | | |

**Table S2: Comparison of the proposed LC–MS/MS method with previously reported LC–MS/MS methods for simultaneous determination of olanzapine and fluoxetine in human plasma.**

| **Reference** | **Plasma Volume** | **Extraction Strategy** | **Solvents in Sample Preparation** | **IS** | **Column Specifications** | **Mobile Phase** | **Flow Rate (mL/min)** | **Run Time (min)** | **Linear Range (ng/mL)** | **Remarks** |
| --- | --- | --- | --- | --- | --- | --- | --- | --- | --- | --- |
| Ni et al. [28] | 0.2 | LLE | Ethyl  acetate, methanol, and NaOH | Stable isotope labeled IS:  Olanzapine‑d3  Fluoxetine‑d5 | Agilent Eclipse plus C_18_ column (4.6 × 100 mm, 5 μm) | Methanol-water containing 20mM ammonium  formate (82.5:17.5, v/v) | 1.0 | 3.3 | OLA: 0.2 -25 ng/mL  FLU: 0.2 -25 ng/mL | Relatively short run time (3.3 min); however, LLE-based extraction involves more complex sample preparation steps compared with simple protein precipitation.  Relatively low fluoxetine recovery (59.96%) reported.  Reliance on isotope-labeled IS increases analytical cost.  Conventional 4.6 mm i.d., 5 µm column operated at 1.0 mL min⁻¹, resulting in higher mobile phase consumption per run (3.3 mL). |
| Fan et al. [29] | 0.2 | SPE | 1% Acetonitrile in  water containing  10mM ammonium  formate (v/v) | Diphenhydramine | Hypersil Gold C18 column  (4.6×150 mm, 5 μm) | Gradient elution. Solvents A and B:  acetonitrile and water containing10 mM  ammonium formate and 0.01% formic acid,  respectively. | 1.0 | 11.0 | OlA: 0.25–50 ng/mL  FLU: 0.5–100 ng/mL | Automated online SPE improves cleanup efficiency; however, it requires specialized and costly valve-switching instrumentation. Longer analysis time (11.0 min).  Conventional 4.6 mm i.d., 5 µm column operated at 1.0 mL min⁻¹, resulting in higher mobile phase consumption per run (11.0 mL). |
| Bonde et al. [30] | 0.2 | SPE | 5% ortho phosphoric  acid in water | Stable isotope labeled IS:  Olanzapine‑d3  Fluoxetine‑d5 | Gold C18 column  (4.6×50 mm, 5 μm) | Methanol-water containing 2mM ammonium  acetate (90:10, v/v) | 1.0 | 2.0 | OlA: 0.1–20 ng/mL  FLU: 0.50–50 ng/mL | Short run time (2.0 min); however, SPE requires multi-step processing and costly cartridges.  Reliance on isotope-labeled IS increases analytical cost.  Conventional 4.6 mm i.d., 5 µm column operated at 0.5 mL min⁻¹, resulting in higher mobile phase consumption per run (2.0 mL). |
| Gopinath et al. [31] | 0.5 | SPE | 2mM ammonium  acetate buffer (pH 8.5, adjusted with ammonia, methanol, solution), 5% methanol in water, 5%  formic acid in acetonitrile | Duloxetine | Hypersil Gold C18 column  (4.6×50 mm, 5 μm) | Acetonitrile–water containing 2% formic acid  (70:30, v/v) | 0.5 | 4.0 | OLA: 0.12–25.03 ng/mL  FLU: 1.00–150.20 ng/mL | Multi-step extraction with costly SPE cartridges.  Longer analysis time.  Conventional 4.6 mm i.d., 5 µm column operated at 0.5 mL min⁻¹, resulting in higher mobile phase consumption per run (2.0 mL). |
| Ravinder et al. [32] | 0.25 | LLE | Methyl tertiary butyl ether and n-hexane (80:20) | Duloxetine | X-terra RP8 column  (4.6×50 mm, 5 μm) | Acetonitrile–water containing 30mM ammonium  formate (90:10, v/v, pH 5.0) | 0.4 | 3.2 | OLA: 0.1–50 ng/mL  FLU: 0.05–25.044 ng/mL | Relatively short run time (3.2 min); however, LLE-based extraction involves multi-step handling.  Conventional 4.6 mm i.d., 5 µm column operated at 0.4 mL min⁻¹, resulting in higher mobile phase consumption per run (1.28 mL). |
| This work | 0.5 | Simple protein precipitation | Acetonitrile | Dextromethorphan | Hypersil Gold column (100 mm × 3 mm, 1.9 μm) column (3 × 100 mm, 1.9 μm) | 0.1% aqueous formic acid and acetonitrile (30:70, v/v) | 0.3 | 2.5 | OLA: 0.2–20.0 ng/mL  FLU: 0.5–50.0 ng/mL for FLU | Simple and rapid protein precipitation sample preparation.  Short run time (2.5 min).  UHPLC 3.0 mm i.d., 1.9 µm column operated at 0.3 mL min⁻¹, resulting in reduced mobile phase consumption per run (0.75 mL), corresponding to a 41.4–93.2% reduction relative to the compared methods.  Unified UHPLC–MS/MS platform for the simultaneous determination of two pharmacologically distinct binary mixtures under identical chromatographic conditions. |

**Table S3: Extraction recovery results (n = 6)**

| **Analyte** | **QC Level** | **Recovery (%)** | **% CV*** |
| --- | --- | --- | --- |
| Mixture 1 | | | |
| DXM | LQC | 92.64 | 1.832 |
|  | MQC | 93.15 | 1.491 |
|  | HQC | 96.73 | 2.576 |
| PSE | LQC | 92.83 | 1.571 |
|  | MQC | 96.85 | 1.912 |
|  | HQC | 92.75 | 1.886 |
| IS | -- | 95.64 | 3.157 |
| Mixture 2 | | | |
| OLA | LQC | 94.56 | 3.526 |
|  | MQC | 95.26 | 2.824 |
|  | HQC | 97.92 | 2.102 |
| FLU | LQC | 92.91 | 1.894 |
|  | MQC | 93.06 | 1.579 |
|  | HQC | 105.70 | 0.892 |
| IS | -- | 102.36 | 2.458 |

**Table S4. System suitability parameters evaluated using six consecutive injections (n = 6) of MQC-level standard solution for all analytes.**

| **Peak Area Ratio %CV** | **Retention Time %CV** | **Analyte/ IS** |
| --- | --- | --- |
| Mixture 1 | | |
| 0.629 | 0.104 | DXM |
| 1.236 | 0.354 | PSE |
| Mixture 2 | | |
| 1.861 | 0.004 | OLA |
| 0.382 | 0.335 | FLU |

**Table S5. AI-assisted results generated by Gemini Pro for the greenness assessment of the proposed LC-MS/MS method using the Analytical GREEnness (AGREE) metric.**

| **AGREE Principle** | **GAC Principle Evaluated** | **Proposed Method Characteristic** | **Calculated Score** | **Color Scale** |
| --- | --- | --- | --- | --- |
| 1 | Sample Treatment | External sample pre-treatment and batch analysis (reduced number of steps). | 0.30 | Orange |
| 2 | Sample Volume | 0.5 mL of human plasma per analysis. | 0.75 | Light Green |
| 3 | Device Positioning | Off-line analysis. | 0.00 | Deep Red |
| 4 | Sample Preparation Steps | ≤ 3 macroscopic steps (solvent addition, vortexing, and centrifugation). | 1.00 | Dark Green |
| 5 | Automation & Miniaturization | Semi-automated autosampler non-miniaturized standard LC-MS/MS system. | 0.25 | Orange |
| 6 | Derivatization | Direct MS detection; no derivatizing agents required. | 1.00 | Dark Green |
| 7 | Waste Generation | 2.25 mL of total analytical waste per sample. | 0.59 | Light Green |
| 8 | Analysis Throughput | 48 analytes determined per hour (24 runs/hour × 2 target analytes/run). | 0.89 | Dark Green |
| 9 | Energy Consumption | High energy consumption (LC-MS/MS system). | 0.00 | Deep Red |
| 10 | Source of Reagents | Aqueous mobile phase component utilized (some reagents from renewable sources). | 0.50 | Yellow |
| 11 | Toxicity | 1.53 mL of toxic/hazardous reagents per sample. | 0.45 | Yellow |
| 12 | Operator Safety | Operator exposed to 1 GHS hazard classes during prep (ACN: flammable) | 0.80 | Green |
|  |  | Overall Composite Score | 0.54 | Yellow |
